# Underlying Subclavian Artery Occlusion Initially Misdiagnosed in Weightlifter Using Anabolic Steroids: A Case Report and Review of Literature

**DOI:** 10.7759/cureus.37763

**Published:** 2023-04-18

**Authors:** Leo Meller, Katherine Wilson, Brady Huang, Sandhya Kalavacherla, Kenneth Vitale

**Affiliations:** 1 Department of Orthopedic Surgery, Division of Sports Medicine, University of California San Diego School of Medicine, La Jolla, USA; 2 Department of Radiology, University of California San Diego School of Medicine, La Jolla, USA

**Keywords:** sports medicine, peripheral artery disease, bodybuilding, anabolic steroid use, thoracic outlet syndrome, subclavian artery occlusion

## Abstract

Subclavian artery occlusion (SAO) is a rare form of peripheral artery disease, sometimes associated with arterial thoracic outlet syndrome (ATOS). Subclavian arterial and venous occlusions are often misdiagnosed initially, and their clinical presentation can be confusing in bodybuilding athletes with increased vascularity in combination with anabolic steroid use. We present a 63-year-old male weightlifter with a history of hypertensive cardiomyopathy, renal transplant with left upper extremity arteriovenous fistula and subsequent takedown, cervical spinal stenosis, left rotator cuff surgery, and decades of testosterone injections who presented with years of left shoulder and neck pain. After having seen multiple providers and being diagnosed with various common disorders, CT angiography and conventional angiography were eventually performed and confirmed the presence of chronic SAO. The chronic occlusion was not deemed amenable to surgery or endovascular intervention and was treated medically with anticoagulation. Although anabolic steroid use is associated with arterial thrombosis, to our knowledge, this is the first reported case of SAO in a weightlifter. Initial misdiagnosis resulted in a long and costly workup. Although the patient’s symptoms were consistent with occlusion (and his increased vascularity could potentially suggest chronic thrombosis of any kind), these key signs were masked given his weightlifting history, anabolic steroid use, and concurrent degenerative musculoskeletal conditions common to the weightlifting population. A thorough history, comprehensive physical examination, appropriate imaging studies, and a high index of suspicion for vascular occlusion in athletes who use steroids are critical for the timely diagnosis and treatment of SAO.

## Introduction

Shoulder and neck pain are estimated to affect between 10% and 26% of the adult population in the United States [1], with shoulder pain being the third most common reason for musculoskeletal consultation in primary care [2]. Common causes of shoulder pain include rotator cuff disorders (i.e., tears, tendinopathy), glenohumeral disorders (i.e., adhesive capsulitis, arthritis), acromioclavicular disease, and nerve impingement syndromes [2]. Neck pain is strongly associated with both psychological and neuromuscular causes, the latter of which includes cervical spondylosis, spinal stenosis, fibromyalgia, and cervical radiculopathy; neck and shoulder pain from anatomic instability or non-orthopedic causes such as arterial thoracic outlet syndrome (ATOS) is rare [3]. Therefore, the possible etiologies for neck and shoulder pain, coupled with the complex anatomy in these regions, create a potential diagnostic challenge in the workup of shoulder and neck symptoms [4]. Misdiagnosis can lead to a prolonged and expensive workup, inappropriate treatment, possible unnecessary testing and/or procedures, and the persistence of pain and symptoms that impair quality of life and limit daily activities.

A rare and often overlooked etiology of shoulder and neck pain is subclavian artery occlusion (SAO). Reported in two percent of the general population and seven percent of individuals with vascular disease [5], SAO is a form of peripheral artery disease (PAD) that can precipitate claudication symptoms (and, in extreme cases, ischemia) to the upper extremity and digits [5].

SAO is most commonly caused by atherosclerosis, with the mechanism involving inflammatory cell adhesion to vascular walls and resultant vessel remodeling and narrowing [5]. Given its anatomical location, SAO can be associated with ATOS, a rare syndrome of varying and overlapping symptoms such as paresthesia, numbness and tingling, temperature changes, and pain sensations in the upper extremity. The incidence of thoracic outlet syndrome itself is considered rare, reported between less than one percent and eight percent, with ATOS even more rare (one to two percent of TOS cases), but may be underdiagnosed [6]. Many routine imaging and electrodiagnostic studies are negative [7], making ATOS essentially a clinical diagnosis of exclusion and difficult to diagnose.

Furthermore, the diagnosis of SAO can be particularly challenging in bodybuilding athletes who use androgenic-anabolic steroids (AAS). On one hand, muscle hypertrophy exercise is known to be cardioprotective by increasing the size and number of existing vessels through sheer stress stimuli and angiogenesis signals [8]. As a result, a patient who exercises regularly could be considered low risk for PAD, and this may lead to a missed diagnosis. Conversely, long-term AAS use has been associated with an increased risk of thrombosis and atherosclerosis [9], causing symptoms consistent with SAO. National estimates place AAS usage prevalence at approximately one percent in the United States; however, the majority of users are bodybuilders and elite athletes [10]. Furthermore, bodybuilders are known to have increased extremity vascularity [8], which may mask signs of venous thrombosis (leading to venous congestion) or arterial occlusion (causing increased arterial and venous collateralization). These changes in vascular physiology present a clinical challenge and potential diagnostic conundrum to clinicians treating these populations.

Altogether, the rarity of SAO, the opposing risks of exercise and AAS use on vascular occlusion, combined with the increased vascularity in bodybuilding athletes and the underappreciation of the incidence of TOS, creates a significant diagnostic challenge for clinicians evaluating AAS-using athletes with shoulder and neck pain. Presently, we describe the first reported case of SAO associated with chronic AAS in a weightlifter, which was initially misdiagnosed as cervical spinal stenosis and glenohumeral arthritis. We further present a literature review of SAO and AAS-induced arterial occlusions.

This article was previously presented as a meeting abstract at the Association of Academic Physiatry 2023 Annual Meeting on February 23, 2023.

## Case presentation

A 63-year-old male weightlifter with a history of hypertensive cardiomyopathy, IgA nephropathy, renal transplant with left upper extremity fistula, cervical spinal stenosis, left rotator cuff surgery, and 40 years of testosterone injections presented to the outpatient sports medicine clinic with 12 years of left shoulder and neck pain, exacerbated by a motorcycle accident five years ago. The patient reported his pain level as 10/10. He described worsening paresthesia and tingling sensations extending down the left arm to his hand, causing difficulty in gripping the motorcycle and driving.

Examination of the patient’s left shoulder revealed significant pain and stiffness, positive crepitus during the range of motion, and an overall limited range of motion in his shoulder. He reported pain and stiffness while holding his arm abducted to 90°, pain and limited range with internal and external rotation, and give-way weakness during rotator cuff testing. He had significant pain and stiffness with flexion and extension of the cervical spine with positive Spurling’s, as well as lateral bending and rotation. The patient was stiffest with cervical spine extension. There were no absent or diminished pulses on exam or history of Adson’s disease; however, the patient presented with increased upper extremity vascularity in the arm, as well as in the neck and shoulder regions bilaterally, considered a common finding in bodybuilders and steroid users. The patient did not have clinical features of Raynaud’s syndrome or upper extremity claudication. He did report chronic shoulder pain with exercise, related to his rotator cuff pathology, but did not have exertional progressive symptoms.

**Figure 1:**
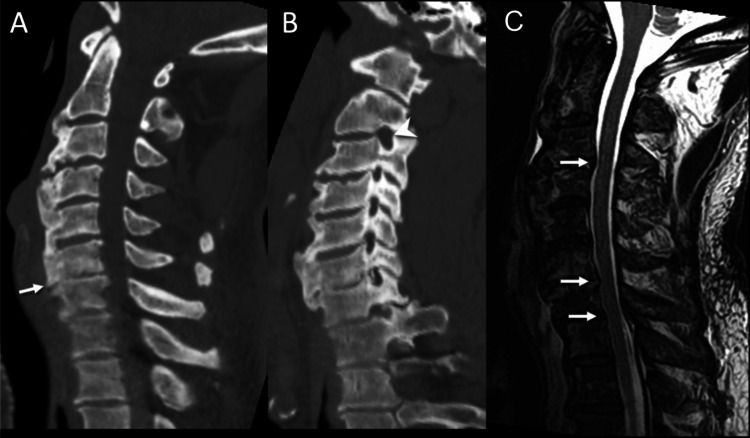
(A) Sagittal cervical spine CT obtained five years prior to the presentation shows multilevel degenerative spondylotic changes, with disc space narrowing, most pronounced at C6-C7 (arrow) with bulky endplate osteophytes. (B) Sagittal oblique multiplanar reconstruction from the CT shows varying degrees of left neural foraminal narrowing in nearly the entire cervical spine, with the exception of the C2-C3 neural foramen (arrowhead). (C) Sagittal T2-weighted MRI of the cervical spine obtained more recently shows varying degrees of spinal canal narrowing due to disc bulges, particularly at the C3-C4, C6-C7, and C7-T1 levels (arrows). CT: computed tomography; MRI: magnetic resonance imaging.

**Figure 2:**
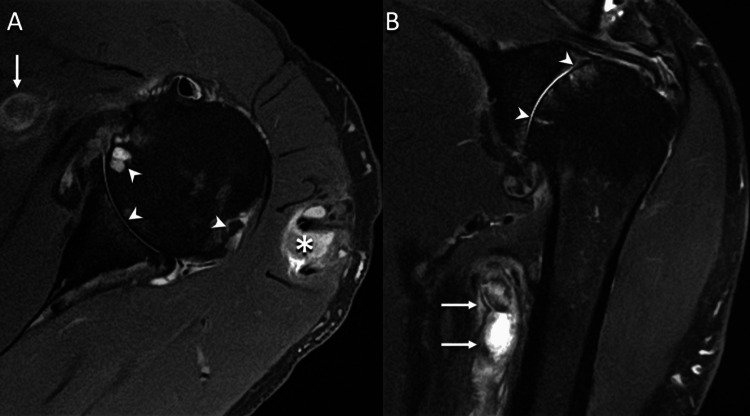
(A) Axial proton density-weighted fat-suppressed and (B) coronal oblique T2-weighted fat-suppressed MR images demonstrate dilated axillary and brachial vessels (arrows) with increased intraluminal signal and perivascular edema. The vessels were initially thought to be veins, which prompted further evaluation with Doppler US. The MR images also show end-stage osteoarthrosis of the glenohumeral joint with global full-thickness chondral loss, subchondral cysts, and marginal osteophytes (arrowheads). There is also a collection (*) within the deltoid muscle related to repeated androgenic-anabolic steroid injections. MR: magnetic resonance; US: ultrasound.

**Figure 3:**
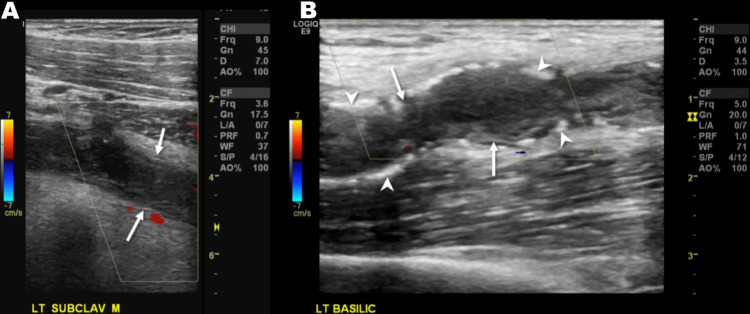
Long axis Doppler US through the (A) upper chest and (B) upper arm performed subsequent to the MRI shows thrombosed vasculature, which was misdiagnosed as thrombosed subclavian and basilic veins, with an echogenic intraluminal thrombus and an absent color Doppler signal (arrows). However, note the echogenic foci within the vessel wall (arrowheads), indicative of atherosclerotic calcifications, which are not typically seen in veins. US: ultrasound; MRI: magnetic resonance imaging.

**Figure 4:**
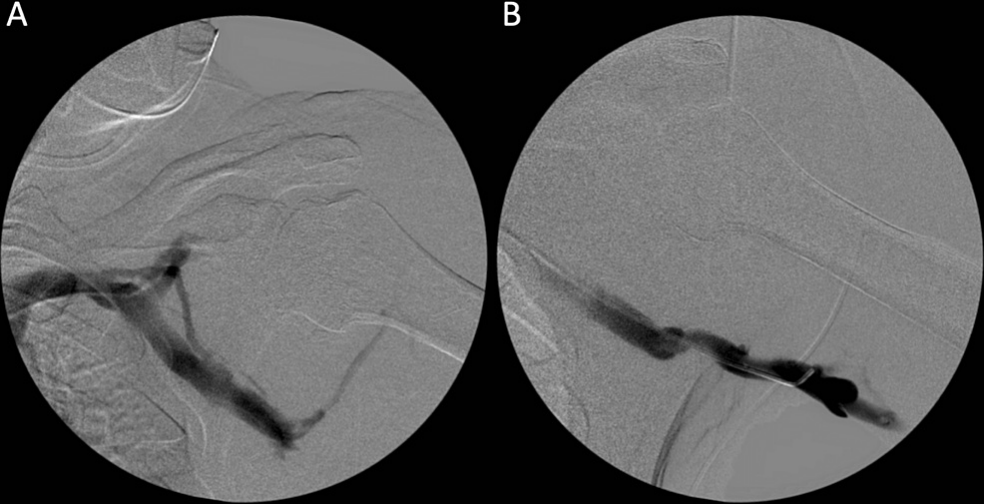
(A) and (B) Venogram of the central and more peripheral portions of the axillary veins opacify with contrast and demonstrate patency.

**Figure 5:**
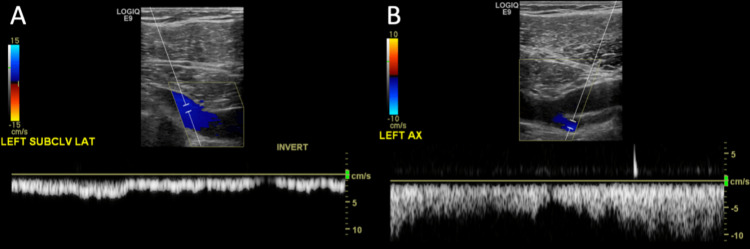
Longitudinal Doppler US of the (A) subclavian and (B) axillary veins demonstrate patent veins with the color flow. US: ultrasound.

**Figure 6:**
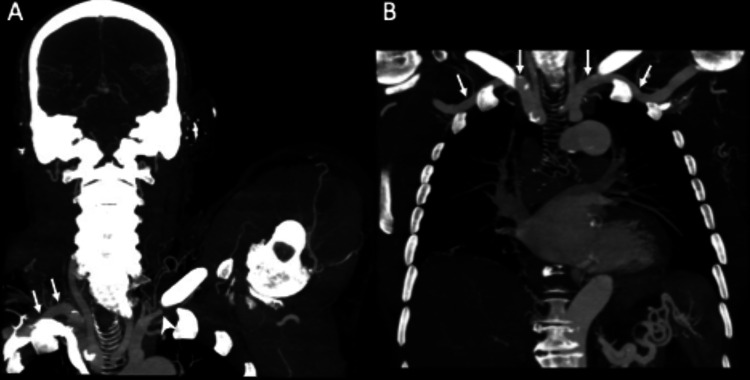
(A) Coronal MIP image (slab thickness 3.0 cm) from a CT angiography of the left upper extremity with the arm raised demonstrates occlusion of the left subclavian artery just central to the costoclavicular space (white arrowhead). The contralateral subclavian artery is patent (arrows). Note the presence of glenohumeral osteoarthritis with humeral head osteophytes and joint space narrowing (black arrowheads). (B) Coronal MIP image from a CT angiography of the chest performed five years earlier in the same patient for trauma shows that both subclavian arteries are patent (arrows), even though both arms are raised during this exam. MIP: maximum intensity projection; CT: computed tomography.

**Figure 7:**
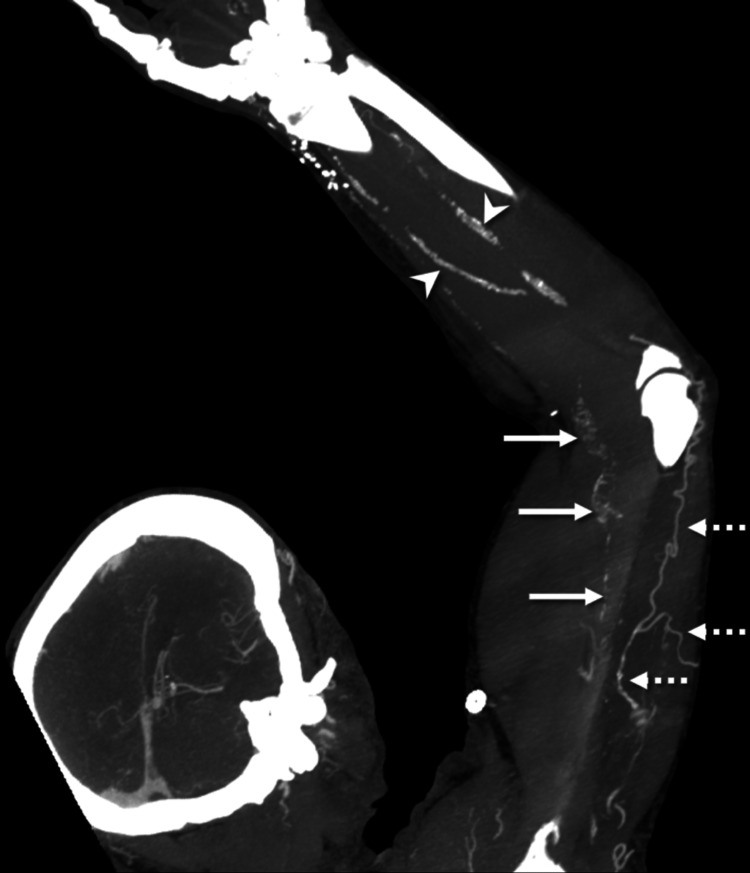
Coronal MIP image (slab thickness 2.0 cm) from a CT angiography of the left upper extremity with the arm raised demonstrates occlusion of the entire brachial artery manifested by the absence of contrast. Note the atherosclerotic calcifications through the brachial artery, corresponding to the prior Doppler US findings (arrows). There is opacification of the downstream radial and ulnar arteries (arrowheads) via an extensive network of collateral vessels (dotted arrows). MIP: maximum intensity projection; CT: computed tomography; US: ultrasound.

**Figure 8:**
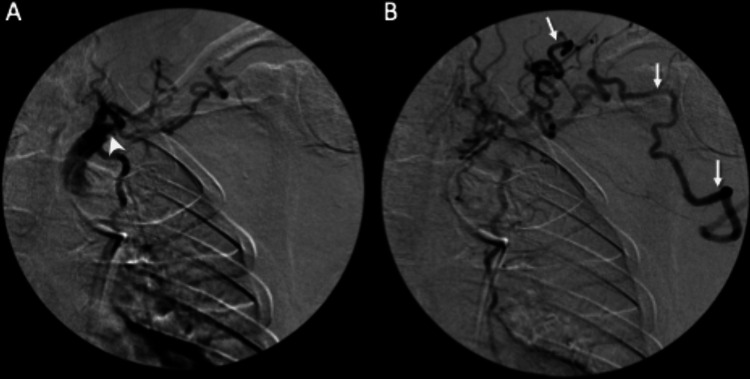
(A) Conventional angiography with the selection of the proximal central subclavian artery shows occlusion (arrowhead) of the subclavian artery as seen on CT angiography of the left upper extremity with collateral vessels. (B) Sequential image shortly after the injection shows serpiginous collateral vessels extending into the arm (arrows). CT: computed tomography.

**Figure 9:**
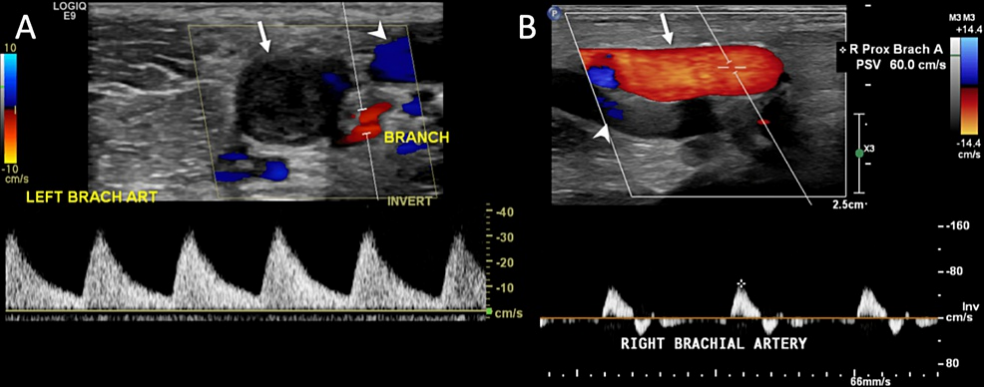
(A) Doppler US of a thrombosed left brachial artery (arrow) shows an echogenic intra-luminal thrombus. Color Doppler shows the absence of flow with the exception of a small side branch adjacent to the artery (BRANCH). Note the abnormal biphasic appearance of the Doppler waveform with spectral broadening with both prolonged upstrokes and downstrokes. Color Doppler shows flow in the adjacent basilic vein (arrowhead). (B) Doppler US of the contralateral patent right brachial artery shows a normal triphasic waveform, with diastolic flow reversal and a small forward flow reflective wave in late diastole. Also, note the small amount of color flow in the adjacent brachial vein (arrowhead). US: ultrasound; BRANCH: branch adjacent to the artery.

In the past, his pain had been presumed to be attributable to his history of cervical spinal stenosis and glenohumeral arthritis. The patient had undergone an extensive workup by multiple providers and tried many conservative measures for these diagnoses, including pain medication, activity modification, physical therapy, working with athletic trainers, and modifications to his workout and exercise program, but he was not satisfied with the degree of symptomatic relief. Due to a lack of response to various conservative measures, further workup was ordered. Due to give-way weakness on testing and a history of prior rotator cuff surgery, shoulder magnetic resonance imaging (MRI) was ordered, as well as a cervical computed tomography (CT) and MRI to assess for radiculopathy. The CT and MRI did redemonstrate significant cervical spinal stenosis (Figures 1A, 1B), glenohumeral arthritis (Figures 2A, 2B), and chronic AAS injections (Figure 2A). However, there were abnormally dilated vessels coursing from the axilla and into the upper arm with altered intra-luminal signal and perivascular signal (Figures 2A, 2B), prompting Doppler imaging. The Doppler imaging was initially misinterpreted as showing thrombosed subclavian and basilic veins but, in retrospect, were indicative of atherosclerotic calcifications (Figures 3A, 3B). A formal venogram (Figures 4A, 4B) and subsequent Doppler ultrasound (Figures 5A, 5B) indicated that the veins were patent. CT angiography and conventional angiography were finally ordered. CT angiography (Figures 6A, 6B) confirmed the left SAO and occlusion of the entire brachial artery (Figures 7A, 7B), with conventional angiography also confirming the left SAO (Figures 8A, 8B). Repeat Doppler ultrasound (Figures 9A, 9B) further demonstrated a left brachial artery thrombus. The patient was ultimately diagnosed with ATOS associated with his chronic AAS use. Vascular surgery consultation deemed the chronic occlusion unamenable to endovascular intervention. Symptoms were ultimately managed with chronic anticoagulants, where the patient completed three months of warfarin (5-7.5 mg range for goal international normalized ratio 2.0-3.0) after consultation by the hematology/oncology and vascular surgery teams. On repeat ultrasound (arterial and venous duplex), no venous thrombosis was noted, but the persistence of a similar and nearly occlusive thrombus was noted in the left subclavian, axillary, and brachial arteries. At three months of treatment, the patient was taken off warfarin for diluted Russell viper venom time (dRVVT) testing for anti-phospholipid antibody syndrome. Hematology consult suggested that if the patient was positive, he would need to resume warfarin. However if negative, the patient could transition to a direct oral anticoagulant (DOAC), as his ongoing testosterone use placed him at risk for recurrent thrombosis (the patient self-reported taking about four times the usual testosterone doses). The dRVVT was negative, and the patient transitioned to apixaban 5 mg twice a day, which continues to this day. In addition, at the follow-up visit about three months after initiation of anticoagulants, the progressive symptoms (for which he had sought treatment) had largely resolved and were back to his baseline (neck pain and shoulder stiffness from the known orthopedic conditions). The patient also had repeat ultrasounds at one month, four months, and eight months since initially put on anticoagulation showing similar thrombus in the left subclavian, axillary, and brachial arteries. Of note, upon long-term follow-up one year from the last ultrasound (totaling one year and eight months since initially starting anticoagulation), another venous ultrasound showed a new but chronically appearing thrombosis of the right basilic vein (otherwise patent and compressible veins); as the patient remains on DOAC, there was no new intervention regarding this finding.

Interestingly, the patient did not have extensive venous thrombosis as initially thought. Certainly, he did have arterial thrombosis as shown by both conventional angiography, CT angiography and Doppler ultrasonography, but this may have been due to the patient’s prior fistula takedown as the veins can be significantly dilated, as a physiologic response to exposure to arterial pressures. Upon further review of the patient’s imaging studies, the “thrombosed veins” on the initial Doppler imaging were incorrectly identified as such, and the thrombosed vessels were actually arterial vessels.

## Discussion

SAO is a very uncommon source of shoulder and neck pain relative to other etiologies. In fact, only 17 cases of SAO have been reported in the literature between 1997 and 2022, among which 82% of cases were initially misdiagnosed (Table 1). Most misdiagnoses led to the persistence of symptoms for months before effective treatment. Specifically, 70% of these SAO cases were caused by bony anomalies of the thoracic outlet, while other causal factors included PAD and smoking.

**Table 1:** Review of published cases of subclavian artery occlusion. PAD: peripheral artery disease; GIB: gastrointestinal bleed; IV: intravenous; COPD: chronic obstructive pulmonary disease.

Case(s)	Case reports	Age/gender	Risk factors	Symptoms, duration	Misdiagnosis?	Treatment	Outcome
1-12	Nehler et al. [11]	17-62/M	Bony abnormalities of the thoracic outlet	Unilateral Reynaud syndrome; exercise-induced fatigue of arm; ischemic pain; ischemic ulceration; average of 7.2 months	Primary vasospastic disorder	Surgical treatment, aneurysm resection, interposition vein grafting with appropriate distal revascularization	Resolution of ischemic symptoms in 11/12 patients
13	Nasrullah et al. [12]	59/F	PAD	Dark stools; dizziness; acute onset	Acute GIB and hemorrhagic shock	Ceasing inappropriate vasopressors	Resolution of symptoms
14	Salman et al. [13]	71/F	None	Cold, painful left arm; 10 days	No	IV heparin, stenting	Resolution of symptoms
15	Salman et al. [13]	59/M	Smoker	Cold left arm; a few years	No	Angioplasty, stenting, carotid subclavian bypass	Resolution of symptoms
16	Salman et al. [13]	64/F	Smoker; COPD; PAD	Left arm claudication; dizziness with left arm movement; unknown duration	No	Angioplasty, carotid-subclavian bypass	Resolution of symptoms
17	Salman et al. [13]	56/F	Smoker	Left arm pain, numbness; acute onset	No	Angioplasty, stenting, carotid-axillary bypass	Resolution of symptoms

It is possible that the effects of bodybuilding and AAS on the vasculature increase the risk of SAO. A review of the literature found five cases between 2009 and 2022 of artery occlusions in bodybuilders using AAS (Table 2). The site of thrombosis, the associated symptoms, and the duration of AAS use all varied across patients. However, no instances of SAO were reported. Hence, to the best of our knowledge, we describe the first case of SAO associated with chronic AAS use in a weightlifter. 

**Table 2:** Review of published cases of artery occlusion in patients who use anabolic steroids. ICU: intensive care unit; IV: intravenous.

Case	Case reports	Age/gender	Steroid usage	Location of occlusion	Symptoms	Treatment	Outcome
1	Damasceno et al. [14]	21/M	Six months	Left supratemporal branch retinal vein occlusion	Rapid onset visual blurring	Ceasing drug usage	Regained complete visual acuity within several months
2	Damasceno et al. [14]	18/M	One year	Left supratemporal branch retinal vein occlusion	Eye floaters	Ceasing drug usage	Regained complete visual acuity within several months
3	Lemiński et al. [15]	34/M	Several months	Renal arteries, bilateral	Severe flank pain	AngioJet rheolytic thrombectomy; continuous renal replacement therapy in ICU	No additional thrombotic events at four-year follow-up
4	Sveinsson et al. [16]	21/M	Four months	Cerebral venous sinus thrombosis	Paresthesia and dyspraxia, left hand; generalized tonic-clonic seizure	IV anticoagulation therapy; oral anticoagulation therapy	Resolution of thrombosis and full recovery at four-month follow-up
5	Tashiro et al. [17]	54/M	Three years	Right coronary artery; subacute stent thrombosis	Chest pain	IV heparin and a temporary pacemaker insertion	Resolution of symptoms

Timely and accurate diagnosis of SAO can be challenging given its rarity. This challenge is compounded in vascular bodybuilding athletes who use AAS, as their enhanced vascularity can mask an underlying occlusion (arterial or venous). In this case, the patient experienced shoulder and neck pain for 12 years, which initially may have been largely orthopedic in nature; however, he underwent thorough workups by multiple providers and tried various conservative treatment attempts before ultimately a diagnosis of SAO was made. Patients with PAD and chronic thrombosis can remain symptomatic without necessarily resulting in necrosis and can remain undiagnosed for months as limb-threatening ischemia occurs in only one to two percent of cases [18]. Until this diagnosis, the patient was treated as if he had cervical spinal stenosis and shoulder arthritis. The average two-year cost of nonoperative management of spinal stenosis is estimated to be $1000 [19]. While there is no specific data on the cost of nonoperative management of glenohumeral arthritis, the Centers for Disease Control and Prevention (CDC) estimates adults with arthritis spend approximately $2000 annually on their medical care [20]. Furthermore, this patient reported frustration in having to see different providers and undergo several rounds of workups and treatments before the eventual SAO diagnosis. Hence, the consequences of SAO misdiagnosis are multifold-not only do patients suffer from the persistence of their symptoms, but they also face an increased financial burden when SAO is mistaken for and managed as a chronic musculoskeletal condition rather than a vascular condition.

The proper evaluation of shoulder and neck pain involves a detailed patient history, physical exam, and, when appropriate, imaging studies. In this case, the patient’s cervical spinal stenosis and shoulder arthritis, as well as past rotator cuff surgery, were misleading to providers, who assumed his symptoms were all from his chronic orthopedic conditions, as is common in bodybuilders, whereas his chronic use of AAS and lack of responsiveness to conservative management of symptoms, along with numbness and tingling down his arm into his hand, should have generated suspicion for a non-musculoskeletal condition.

With vascular occlusion, patients may report a variety of nonspecific symptoms, including muscle fatigue, tingling, aching, burning, cramping, and pain, which may overlap with orthopedic conditions. Less frequently are the more telling overt ischemic symptoms of finger ulcers and necrosis observed [5]. Duplex ultrasound with color flow imaging is the preferred noninvasive modality for evaluating SAO [5]. Signs of SAO can be indicated by weak upper extremity pulses and, classically, a blood pressure difference of more than 15 mmHg between the affected and unaffected arms [5]. In patients with bilateral SAO, blood pressure values in the lower extremities need to be considered, and an ankle-brachial index greater than 1.3 is suggestive of bilateral SAO. A definitive diagnosis is ultimately made through invasive angiography [5].

This patient’s SAO diagnosis was complicated by his history of bodybuilding and AAS use, as the enhanced vascularity from AAS use and bodybuilding can mask the prominent neck, shoulder, and upper extremity vascularity associated with chronic arterial and vascular occlusions and the associated vascular congestion and collateralization. In our patient, this was further complicated by his history of an upper extremity fistula in the same arm, followed by takedown surgery, which can result in significant vein dilation from the increased pressures. The masking of vascular occlusion symptoms is dangerous due to necrosis and thromboembolic events. As a result, underlying vessel occlusions should be highly considered in any patient presenting with symptoms suggestive of SAO. If patient history, physical exam, and imaging findings suggest symptoms originate from a non-musculoskeletal source, providers should have a strong suspicion to consider a vascular explanation and have a low threshold for initiating a workup. Ultimately, appropriate imaging is critical for timely diagnosis and proper treatment of SAO and other occlusions, which can present similarly to more common musculoskeletal disorders. 

## Conclusions

We report the first case of chronic steroid-induced SAO in a vascular weightlifter experiencing neck and shoulder pain with worsening paresthesia and tingling to his hand, who was initially misdiagnosed with cervical spinal stenosis and shoulder arthritis and suffered a costly and long workup. SAO is often misdiagnosed given its rarity, and the associated ATOS can underlie musculoskeletal pain of the upper extremity and head and neck. In patients presenting with symptoms consistent with ATOS, clinicians should consider underlying SAO in patients with AAS use and remain aware that the increased vascularity induced by bodybuilding and AAS may mask potential occlusions.
